# STAT6 Deficiency Accelerates DMBA/TPA-Induced Skin Papilloma Development and Is Associated with Regulatory T-Cell Accumulation and Reduced CD8+IFN-γ+ Responses

**DOI:** 10.3390/biology15191747

**Published:** 2026-10-02

**Authors:** Aranza Mejía-Muñoz, Tania Galindo-Santamaría, Valeria Moreno-Tovar, Citlaltepetl Salinas-Lara, Carlos Sánchez-Garibay, Rubén D. Arroyo Olarte, Sonia Leon-Cabrera

**Affiliations:** 1 Unidad de Biomedicina (UBIMED), Facultad de Estudios Superiores-Iztacala, Universidad Nacional Autónoma de México, Tlalnepantla 54090, Mexico; aranzamar6@gmail.com (A.M.-M.); ruben.arroyo@iztacala.unam.mx (R.D.A.O.); 2 Carrera de Médico Cirujano, Facultad de Estudios Superiores-Iztacala, Universidad Nacional Autónoma de México, Tlalnepantla 54090, Mexico; 3 Neuropatología, Instituto Nacional de Neurología y Neurocirugía Manuel Velasco Suárez, Mexico City 14269, Mexico

**Keywords:** STAT6, DMBA/TPA-induced skin carcinogenesis, regulatory T cells, Foxp3, CD8+IFN-γ+ T cells, tumor immune microenvironment, cutaneous squamous carcinogenesis, immune surveillance, papilloma development

## Abstract

Inflammation and the balance between antitumor surveillance and immunoregulatory mechanisms influence chemically induced skin tumor development. STAT6 is a central mediator of IL-4/IL-13 signaling, but its contribution to early skin papilloma development remains unclear. Here, we examined papilloma onset and burden, histopathological alterations, Treg accumulation, and CD8+ T-cell-associated responses in WT and STAT6-deficient mice subjected to a two-stage DMBA/TPA protocol. STAT6−/− mice developed papillomas significantly earlier and exhibited a markedly greater lesion burden than WT mice. This phenotype was associated with more prominent keratinization-related histopathological features, increased Foxp3+ Treg frequency and PD-1 expression in skin-draining lymph nodes, and a broader distribution of Foxp3+ cells within papillomas. STAT6 deficiency was also accompanied by attenuated CD8+IFN-γ+ responses in draining lymph nodes and reduced CD8+ cell infiltration within lesions. Collectively, these findings reveal a distinct immune profile associated with enhanced susceptibility to chemically induced skin papillomas and underscore the context-dependent role of STAT6 in cutaneous tumor immunity.

## Simple Summary

Skin tumor development is strongly influenced by inflammation and immune responses within the affected tissue. In this study, we investigated the role of STAT6, a protein involved in immune regulation, in a mouse model of chemically induced skin papilloma development. Mice lacking STAT6 developed papillomas earlier and in greater numbers than wild-type mice. This increased papilloma burden was associated with greater accumulation of regulatory T cells and reduced CD8+ T-cell infiltration and IFN-γ expression. Together, these findings identify STAT6 as an important component of the immune environment associated with susceptibility to skin papilloma development. This study highlights the context-dependent role of STAT6 in tumor development and suggests that therapeutic strategies targeting this pathway should consider tissue-specific immune effects.

## 1. Introduction

Signal transducer and activator of transcription 6 (STAT6) is a cytoplasmic transcription factor that translocates to the nucleus upon phosphorylation. STAT6 is activated primarily downstream of interleukin-4 (IL-4) and interleukin-13 (IL-13). IL-4 signals through receptor complexes containing IL-4Rα, whereas IL-13 signals predominantly through the type II receptor composed of IL-4Rα and IL-13Rα1. Ligand binding activates Janus kinases (JAKs) and tyrosine kinase 2 (TYK2), creating docking sites for STAT6. STAT6 is subsequently phosphorylated, forms homodimers, and translocates to the nucleus, where it regulates the transcription of genes involved in cell survival, inflammation, proliferation, and immune regulation [1,2].

STAT6 is widely recognized as a central mediator of type 2 immune responses. It has also been implicated in tumor progression across several malignancies, including prostate cancer, melanoma, glioblastoma, colorectal cancer, cutaneous T-cell lymphomas, and other hematologic malignancies [3,4,5,6,7]. Persistent epithelial damage promotes the release of inflammatory mediators, immune-cell recruitment, and remodeling of the local tissue microenvironment. In this context, the balance between antitumor effector responses and immunoregulatory mechanisms determines whether transformed cells are eliminated or allowed to progress [8,9]. Regulatory T cells (Tregs), characterized by expression of the transcription factor Foxp3, are central mediators of immune tolerance and tissue homeostasis. However, within the tumor microenvironment (TME), their accumulation can suppress cytotoxic antitumor responses and favor immune escape [10].

Several studies suggest that STAT6 may regulate Treg biology through direct or indirect effects on Foxp3 expression. STAT6 has been described as a global regulator of gene expression during T-helper-cell differentiation and has been reported to repress Foxp3 transcription [11]. Chromatin immunoprecipitation assays identified a silencer region within the Foxp3 locus that contains a specific STAT6-binding site and prevents Foxp3 mRNA expression [12]. In addition, IL-4, a cytokine that activates STAT6, significantly reduces TGF-β-induced differentiation of CD4+ T cells into CD4+Foxp3+ cells in vitro [13]. This evidence suggests that STAT6-dependent signaling can influence Foxp3 induction and Treg differentiation.

Consistent with these observations, our previous studies identified STAT6 as an important regulator of Treg-associated responses in colitis-associated colorectal cancer (CAC) [14]. STAT6-deficient mice are highly resistant to CAC development and show increased frequencies of CD4+Foxp3+CD25+ cells in the colon, peripheral blood, and spleen, together with increased expression of TGF-β, IL-10, and Foxp3 compared with wild-type (WT) mice [14,15]. The results indicate that STAT6 influences Treg-associated responses in inflammation-driven cancer. However, whether STAT6 is similarly associated with changes in Treg accumulation in other epithelial tumor models, particularly during cutaneous carcinogenesis, remains unclear.

Several lines of evidence suggest that signaling components upstream of STAT6 may influence skin carcinogenesis. The two-stage 7,12-dimethylbenz[a]anthracene (DMBA)/12-O-tetradecanoylphorbol-13-acetate (TPA) model is widely used to study inflammation-associated skin tumor development. During the initial stages of this model, mice predominantly develop benign papillomas, some of which may undergo malignant conversion to squamous cell carcinoma after extended follow-up [16]. Previous knockout studies have shown that mice lacking IL-4Rα or IL-13Rα1 develop papillomas earlier and in greater numbers than WT animals following DMBA/TPA treatment [17]. Moreover, IL-13Rα1-deficient mice show increased Treg numbers during papilloma development, suggesting that type 2 cytokine receptor signaling may influence the immunoregulatory landscape associated with skin papillomas [18].

Interpreting IL-4 and IL-13 biology in skin carcinogenesis is complex because both cytokines share receptor components and converge on STAT6 activation. In addition, the absence of one ligand or receptor chain may be partially compensated by alternative receptor usage or overlapping downstream signaling pathways. Studying STAT6 directly therefore provides an opportunity to evaluate a common transcriptional node influenced by IL-4 and IL-13, as well as by other potential canonical and noncanonical inputs. This is particularly relevant because specific STAT6 inhibitors are currently being investigated as therapeutic tools [19], and STAT6 activity may influence the immune composition of the tissue microenvironment, including Treg accumulation.

In human skin tumors, STAT3, STAT5A, STAT5B, and STAT6 expression has been reported across histopathological subtypes of basal cell carcinoma (BCC), suggesting that STAT family members may contribute to keratinocyte-derived malignancies [20,21]. However, the role of STAT6 in cutaneous squamous tumor development remains unclear.

In the DMBA/TPA-induced skin carcinogenesis model, T-lymphocyte subsets can affect tumor development differently. CD8+ T lymphocytes generally exert protective functions, whereas certain CD4+ T-cell populations can promote tumor progression [22]. These effects are influenced, in part, by the cytokine profiles and functional properties of each subset. Interferon-gamma (IFN-γ), an effector cytokine produced by activated CD8+ T cells and other immune populations, can enhance cytotoxic T-cell activity, promote apoptosis in transformed cells, and modulate immune-cell trafficking within the tissue microenvironment [23]. IFN-γ production by CD8+ T cells has also been associated with improved responses to cancer immunotherapy [24]. Nevertheless, IFN-γ effects can vary by cellular source, timing, and inflammatory context. Therefore, evaluating both Foxp3+ Tregs and CD8+IFN-γ+ T cells may provide insight into the regulatory and effector immune profiles associated with skin papilloma development.

Therefore, we investigated the contribution of STAT6 to the early stages of chemically induced skin papilloma development using the DMBA/TPA model. By integrating longitudinal papilloma monitoring with histopathological, transcriptional, immunohistochemical, and flow-cytometric analyses, we characterized Treg accumulation and CD8-associated immune alterations accompanying STAT6 deficiency.

## 2. Materials and Methods

### 2.1. Animals

Eight- to ten-week-old wild-type (WT) and STAT6-deficient Foxp3-EGFP reporter mice on a BALB/c genetic background were used. In these mice, the Foxp3 locus drives expression of enhanced green fluorescent protein (EGFP), enabling identification of Foxp3-expressing cells by flow cytometry. STAT6−/− Foxp3-EGFP mice were generated and maintained as previously described [14]. WT and STAT6−/− Foxp3-EGFP mice were maintained as separate homozygous colonies and were therefore not littermate-derived. To minimize potential confounding factors, animals were age- and sex-matched, housed in the same animal facility, and maintained under identical environmental and husbandry conditions.

Animals were bred and housed under controlled environmental conditions at the animal facility of the Facultad de Estudios Superiores Iztacala, Universidad Nacional Autónoma de México (UNAM), with food and water provided ad libitum. We randomly assigned mice within each genotype to untreated control or DMBA/TPA-treated groups. All animal procedures were conducted in accordance with institutional animal-care guidelines and were approved by the Bioethics Committee for Animal Research of the Facultad de Estudios Superiores Iztacala, UNAM (approval no. CE/FESI/012022/1456).

### 2.2. DMBA/TPA-Induced Papilloma Model

Skin papilloma development was induced using a two-stage DMBA/TPA protocol. Forty-eight hours before carcinogen administration, the dorsal skin of mice was shaved under anesthesia with xylazine (7 mg/kg) and ketamine (100 mg/kg) to minimize skin injury during hair removal.

On day 1, mice received a single topical application of 50 µg of 7,12-dimethylbenz[a]anthracene (DMBA) (Sigma-Aldrich, St. Louis, MO, USA) dissolved in 200 µL of acetone to the shaved dorsal skin. Seven days after DMBA initiation, tumor promotion began with topical application of 5 µg of 12-O-tetradecanoylphorbol-13-acetate (TPA) (Sigma-Aldrich, St. Louis, MO, USA); dissolved in 200 µL of acetone. TPA was applied twice weekly for 19 consecutive weeks, completing a 20-week DMBA/TPA initiation–promotion skin carcinogenesis protocol.

### 2.3. Tumor Monitoring and Tissue Collection

Two independent experiments were conducted, with five mice per group in each experiment. Papilloma number and diameter were recorded weekly for 20 weeks, along with standardized dorsal photographs. Individual lesions were mapped and tracked longitudinally. A palpable lesion greater than 1 mm in diameter, detected at two consecutive weekly examinations, was considered a papilloma, based on previously established criteria with minor modifications [16].

The mouse was considered the experimental unit. Multiple lesions from the same animal were treated as nested observations and were not treated as independent biological replicates. When indicated, data from both experiments were combined, yielding 10 mice per group.

Animal welfare and body weight were monitored weekly. Humane endpoints included body weight loss greater than 15%, a papilloma exceeding 10 mm, ulceration, persistent bleeding, secondary infection, systemic distress, or deterioration in general condition. Animals were euthanized by sevoflurane (Baxter Healthcare Corporation, Deerfield, IL, USA) inhalation at week 20 or earlier if any humane endpoint was reached.

Dorsal skin and papilloma tissues were collected for histological, immunohistochemical, and gene expression analyses. Skin-draining axillary and inguinal lymph nodes were collected for flow cytometry.

### 2.4. Histology and Histopathological Lesion Analysis

Papilloma tissues and healthy skin samples were fixed in 4% formaldehyde for 24 h at room temperature, dehydrated through graded ethanol solutions, cleared in xylene, and embedded in paraffin. Serial sections (3 µm) were stained with hematoxylin and eosin (H&E) using standard procedures.

H&E-stained sections were evaluated using a predefined semiquantitative scoring system comprising nine histopathological features: acanthosis, hyperkeratosis, parakeratosis, atypia, inflammatory infiltrate, subcutaneous edema, keratin pearl-like structures, vasculitis, and adnexal damage. Each feature was graded as 0 (absent), 1 (mild or focal), 2 (moderate or multifocal), or 3 (severe or diffuse), as detailed in Appendix A, Table A2. Images were acquired using an AxioCam MRc camera (Carl Zeiss Microscopy GmbH, Jena, Germany) and ZEN lite software (version 2011; Carl Zeiss Microscopy GmbH, Jena, Germany). All available papillomas from 10 mice per genotype, obtained from two independent experiments, were evaluated. Three observers blinded to genotype examined four representative microscopic fields per lesion, and resolved discrepancies by consensus. Field-level scores were averaged within each lesion, and lesion-level scores were subsequently averaged to obtain a single biological value per mouse. Thus, the mouse was the experimental unit, and individual fields and papillomas were not treated as independent biological replicates.

Epidermal thickness was quantified from H&E-stained sections using ImageJ (version 1.54t; National Institutes of Health, Bethesda, MD, USA). The complete cross-sectional area of the epidermis was manually delineated, and the corresponding basement membrane length was measured. Mean epidermal thickness was calculated by dividing the epidermal area by the basement membrane length, thereby accounting for variations in epidermal contour and skin undulations. When multiple lesions were available from the same animal, lesion-level measurements were averaged to generate a single biological value per mouse. Thus, the mouse was considered the experimental unit. The analysis included 10 mice per genotype from two independent experiments.

### 2.5. Immunohistochemistry

Paraffin-embedded tissue sections (3 µm) were used to detect Ki-67, Foxp3, and CD8 by immunohistochemistry. Sections were deparaffinized at 59 °C for 30 min, cleared in xylene, and rehydrated through graded ethanol solutions. Antigen retrieval was performed using Diva Decloaker (1:10; Biocare Medical, Pacheco, CA, USA) for 30 min using a pressure cooker. After cooling, sections were washed with PBS containing 0.05% Tween-20 and permeabilized with 0.1% Triton X-100 for 30 min.

Nonspecific binding was blocked with 2% bovine serum albumin (BSA). Sections were incubated overnight at 4 °C with antibodies against Ki-67 (1:100), Foxp3 (1:50), or CD8 (1:50) all purchased from GeneTex (Irvine, CA, USA) diluted in PBS containing 1% BSA, followed by incubation with the corresponding secondary antibody for 1 h at room temperature. Immunoreactivity was visualized using 3,3′-diaminobenzidine (DAB). Sections were counterstained with hematoxylin, dehydrated, cleared, and mounted with Entellan (Merck KGaA, Darmstadt, Germany).

Images were acquired using an AxioCam MRc camera (Carl Zeiss Microscopy GmbH, Jena, Germany) and ZEN lite 2011 software (version 2011; Carl Zeiss Microscopy GmbH, Jena, Germany). For Ki-67 analysis, the percentage of DAB-positive area was determined in three representative fields per mouse using the color-segmentation tool in ImageJ (version 1.54t; National Institutes of Health, Bethesda, MD, USA). For CD8 analysis, CD8-positive cells were counted in three representative fields per mouse. Measurements from the three fields were averaged to obtain a single value per animal; thus, the mouse was considered the experimental unit, and each point in the corresponding graphs represents one mouse. Four mice per group from one representative experiment were included in the quantitative Ki-67 and CD8 analyses. Comparable staining patterns were observed in two independent experiments.

### 2.6. Flow Cytometry

Skin-draining axillary and inguinal lymph nodes were collected from each mouse, pooled, and processed to prepare a single-cell suspension for flow-cytometric analysis. Lymph nodes were mechanically dissociated in sterile saline or RPMI medium. Cell suspensions were centrifuged, washed, and resuspended in FACS buffer. Approximately 1 × 10^6^ cells per sample were analyzed by flow cytometry.

For surface staining, Fc receptors were blocked with anti-CD16/CD32 antibody (BioLegend, San Diego, CA, USA) for 15 min at 4 °C to prevent nonspecific antibody binding. Cells were then stained with Zombie NIR viability dye (BioLegend, San Diego, CA, USA) and fluorochrome-conjugated antibodies against surface markers, including CD4, CD8, and PD-1 all purchased from BioLegend (San Diego, CA, USA) for 30 min at 4 °C in the dark.

For intracellular IFN-γ detection, lymph node cells were polyclonally stimulated with phorbol 12-myristate 13-acetate (PMA; 25 ng/mL) and ionomycin (0.5 µg/mL) in the presence of monensin (2 mM) all purchased from Thermo Fisher Scientific (Waltham, MA, USA) for 4–6 h at 37 °C and 5% CO_2_ to prevent cytokine secretion. After stimulation, cells were centrifuged at 300× *g* for 10 min at 4 °C, then resuspended in FACS buffer for surface and viability staining. Cells were fixed with 4% paraformaldehyde in Dulbecco’s phosphate-buffered saline (DPBS) for 20 min at 4 °C, washed with permeabilization buffer (DPBS supplemented with 0.025% saponin and 1% FBS), and stained intracellularly with APC-conjugated anti-mouse IFN-γ antibody diluted in permeabilization buffer for 10 min at room temperature. Finally, cells were washed and resuspended in 350 µL of DPBS for flow cytometric acquisition.

Lymphocytes were identified based on forward- and side-scatter characteristics, followed by singlet discrimination and exclusion of dead cells using Zombie NIR (BioLegend, San Diego, CA, USA). Viable lymphocytes were subsequently gated according to CD4 or CD8 expression. At least 5000 CD4+ or CD8+ events were included in the respective analyses. Fluorescence-minus-one and unstained controls were used to establish the positivity thresholds for CD4, CD8, PD-1, and IFN-γ expression. Foxp3-expressing cells were identified based on endogenous EGFP fluorescence, and the Foxp3-EGFP-positive threshold was established using reporter-negative controls. CD4+Foxp3-EGFP+ Tregs were identified within the viable CD4+ population, and PD-1 expression was evaluated within the CD4+Foxp3-EGFP+ population. IFN-γ expression was analyzed within the viable CD8+ T-cell population. Samples were acquired using an Attune NxT flow cytometer (Thermo Fisher Scientific, Waltham, MA, USA), and data were analyzed using FlowJo software (version 11.2; Tree Star, Inc., Ashland, OR, USA).

### 2.7. RNA Extraction and Quantitative Real-Time PCR

Because Foxp3+ Tregs represented a low-abundance population within papilloma tissues and isolation of sufficient numbers of these cells for RNA analysis was technically limiting, gene expression was evaluated using RNA extracted from whole papilloma tissue. Total RNA was extracted from papilloma tissues using the All-In-One DNA/RNA/Protein Miniprep Kit (Bio Basic inc, Markham, ON, Canada) according to the manufacturer’s instructions. RNA concentration and purity were assessed by measuring absorbance at 260 and 280 nm using an Epoch™ microplate spectrophotometer (BioTek Instruments, Winooski, VT, USA), and samples with an A260/A280 ratio > 1.8 were used for subsequent analyses.

A total of 40–100 ng of RNA was reverse-transcribed using the RevertAid First Strand cDNA Synthesis Kit (Thermo Fisher Scientific, Waltham, MA, USA) according to the manufacturer’s instructions. Quantitative real-time PCR was performed using 1 µL of cDNA, SYBR Green qPCR Master Mix (MedChemExpress, Monmouth Junction, NJ, USA), and gene-specific primers on a CFX96 Touch™ Real-Time PCR Detection System (Bio-Rad, Hercules, CA, USA). Primer sequences are listed in Appendix A, Table A1.

Relative mRNA expression was calculated using the 2^−ΔΔCt^ method, with 18S rRNA as the reference gene. The selection of 18S rRNA was supported by its consistent amplification across the analyzed samples, with Ct values ranging from 15 to 18 cycles under all experimental conditions.

### 2.8. Statistical Analysis

Statistical analyses were performed using GraphPad Prism (Version 8; GraphPad Software, Boston, MA, USA) and R software (Version 4.6.0; R Foundation for Statistical Computing, Vienna, Austria). For continuous datasets analyzed using parametric tests, normality and homogeneity of variance were assessed using the Shapiro–Wilk and Brown–Forsythe tests, respectively.

Longitudinal papilloma counts from two independent experiments were analyzed jointly using a generalized linear mixed-effects model (GLMM) with a Poisson distribution and log link function. Genotype (WT versus STAT6−/−), week, and their interaction were included as fixed effects. Mouse identity was included as a random intercept to account for repeated measurements and within-animal correlations over time. All animals were included in the analysis, including those that remained papilloma-free.

Model adequacy was evaluated using standardized simulated residuals generated with the DHARMa package. Overdispersion was assessed using a nonparametric dispersion test based on residual standard deviation, zero inflation was evaluated by comparing the observed and model-expected numbers of zeros, and residual uniformity was assessed using the Kolmogorov–Smirnov test. Residual quantiles were also visually inspected against predicted values. Model-derived genotype contrasts at individual weeks were obtained using estimated marginal means. The GLMM and post hoc comparisons were performed in R using the lme4, DHARMa, and emmeans packages.

Time to papilloma onset was analyzed using Kaplan–Meier curves and the log-rank (Mantel–Cox) test. Comparisons involving more than two independent groups were performed using one-way ANOVA followed by Tukey’s multiple-comparisons test. Comparisons between two independent groups were performed using an unpaired two-tailed Student’s t-test. Statistical significance was defined as *p* < 0.05. Data are presented as mean ± SEM unless otherwise indicated. For quantitative endpoint analyses, each data point represents one mouse.

## 3. Results

### 3.1. STAT6 Deficiency Is Associated with Earlier Papilloma Onset and Increased Papilloma Burden

To determine whether STAT6 influences DMBA/TPA-induced skin papilloma development, STAT6−/− mice and WT controls were subjected to a two-stage initiation–promotion protocol. A single topical application of DMBA was administered to the dorsal skin, followed one week later by twice-weekly topical applications of TPA for 19 weeks (Figure 1A). We monitored papilloma development throughout the 20-week experimental period. The first papillomas were detected at week 5 in STAT6−/− mice, and all STAT6−/− animals had developed papillomas by week 12. In contrast, papillomas were first detected at week 10 in WT mice, and only 8 of 10 WT animals (80%) had developed papillomas by week 20. Kaplan–Meier analysis confirmed significantly earlier papilloma onset in STAT6−/− mice than in WT mice (log-rank [Mantel–Cox] test, *p* = 0.0016; Figure 1B).

Longitudinal analysis using a generalized linear mixed-effects model with a Poisson distribution showed a significantly higher papilloma burden in STAT6−/− mice than in WT controls (Figure 1C). At week 20, STAT6−/− mice presented 17.0 ± 2.5 papillomas per mouse, compared with 1.3 ± 0.3 in WT mice (Figure 1D). Overall, 169 papillomas were recorded in STAT6−/− mice and 13 in WT mice. Their distribution according to diameter category is presented descriptively in Figure 1E, and representative images of the dorsal skin are shown in Figure 1F. Because each papilloma was individually mapped and tracked longitudinally, the increased burden in STAT6−/− mice reflected a greater number of distinct persistent lesions rather than repeated counting of transient lesions. However, their earlier onset also provided a longer interval for papilloma accumulation before the common week-20 endpoint.

### 3.2. STAT6 Deficiency Is Associated with Distinct Histopathological Alterations During Papilloma Development

To characterize the histopathological alterations associated with papilloma development, H&E-stained sections of healthy skin and DMBA/TPA-treated tissues from WT and STAT6−/− mice were evaluated. DMBA/TPA treatment induced marked epidermal alterations in both genotypes (Figure 2A,B). Papillomas from WT and STAT6−/− mice exhibited nucleated cells within the stratum corneum, consistent with parakeratosis, together with focal detachment of the stratum corneum. Descriptive semiquantitative scoring showed numerically higher mean scores for acanthosis, subcutaneous edema, hyperkeratosis, and keratin pearl-like structures in STAT6−/− papillomas than in WT lesions (Figure 2B). These observations indicate more prominent keratinization-related features in STAT6−/− papillomas but do not establish malignant transformation or enhanced squamous differentiation.

Epidermal thickness was significantly increased in DMBA/TPA-treated tissues compared with healthy control skin. However, no significant difference was detected between DMBA/TPA-treated WT and STAT6−/− mice (Figure 2C). Ki-67 immunohistochemistry similarly showed an increased Ki-67-positive area in DMBA/TPA-treated tissues compared with healthy skin. At week 20, the Ki-67-positive area was higher in WT lesions than in STAT6−/− lesions (Figure 2D,E).

Collectively, these findings show that DMBA/TPA treatment induced substantial epidermal remodeling in both genotypes. Descriptive histopathological assessment identified more prominent keratinization-related features in STAT6−/− papillomas. Because tissues were examined only at the week-20 endpoint, the present analysis cannot determine whether the difference in Ki-67-positive area reflects lesion age, differentiation state, or another genotype-dependent effect.

### 3.3. STAT6 Deficiency Is Associated with Increased Treg Accumulation in Skin-Draining Lymph Nodes and Papillomas

Previous work from our group demonstrated that STAT6 deficiency promotes PD-1 expression in Tregs and is associated with enhanced suppressive activity in inflammation-associated cancer models [14]. Based on these findings, PD-1 was selected as a phenotypic marker to examine whether Treg accumulation in STAT6−/− mice was accompanied by a similar immunoregulatory profile during DMBA/TPA-induced skin papilloma development. We therefore analyzed CD4+Foxp3+ Tregs and PD-1-expressing CD4+Foxp3+ Tregs in draining axillary and inguinal lymph nodes by flow cytometry. Figure 3A shows the corresponding gating strategy. After 20 weeks of DMBA/TPA treatment, the frequency of CD4+Foxp3+ Tregs was significantly higher in STAT6−/− mice than in DMBA/TPA-treated WT mice and healthy controls (18.4 ± 1.1% in STAT6−/− DMBA/TPA mice versus 7.1 ± 1.1% in WT DMBA/TPA mice, 10.3 ± 0.1% in healthy WT mice, and 13.7 ± 0.1% in healthy STAT6−/− mice; Figure 3B,C). Consistently, the absolute number of CD4+Foxp3+ Tregs in draining lymph nodes was significantly increased in DMBA/TPA-treated STAT6−/− mice compared with the other experimental groups (Figure 3D).

The frequency of PD-1+ cells within the CD4+Foxp3+ Treg compartment was also significantly higher in DMBA/TPA-treated STAT6−/− mice than in DMBA/TPA-treated WT mice and healthy controls (Figure 3E–G). Although the absolute number of PD-1+ Tregs increased numerically in DMBA/TPA-treated STAT6−/− mice, this difference did not reach statistical significance. These findings demonstrate increased accumulation of Foxp3+ Tregs with a PD-1-enriched phenotype in the draining lymph nodes of STAT6−/− mice. However, PD-1 expression alone does not establish enhanced suppressive activity, which would require functional assessment.

We next investigated whether the increased Treg accumulation observed in draining lymph nodes was also present locally within papillomas. Foxp3+ cells were evaluated by immunohistochemistry. Representative immunohistochemical images suggested a broader distribution of Foxp3+ cells within STAT6−/− papillomas, whereas Foxp3+ cells were scarce within WT papillomas and were detected predominantly around hair follicles in adjacent skin (Figure 3H). These findings indicate that STAT6 deficiency is associated with increased accumulation of Foxp3+ cells in both draining lymph nodes and papilloma tissues.

To further characterize the local immunoregulatory profile, we measured the expression of the Treg-associated genes *Foxp3*, *Pdcd1*, and *Ctla4* in whole papilloma tissues. We analyzed whole lesions because Foxp3+ Tregs were a low-abundance population and could not be recovered in sufficient numbers for cell-specific RNA analysis.

Foxp3 expression was increased in papillomas from both WT and STAT6−/− mice relative to their respective healthy skin controls. Moreover, STAT6−/− papillomas exhibited significantly higher Foxp3 mRNA levels than WT papillomas. Interestingly, healthy STAT6−/− skin also showed higher basal *Foxp3* expression than healthy WT skin (Figure 3I).

Similarly, Pdcd1 expression was increased in papillomas from both genotypes compared with healthy controls. Healthy STAT6−/− skin also exhibited higher basal *Pdcd1* expression than healthy WT skin; however, no significant difference was detected between WT and STAT6−/− papillomas (Figure 3J). In contrast, *Ctla4* expression did not differ between healthy and DMBA/TPA-treated WT tissues but was significantly reduced in STAT6−/− papillomas relative to healthy STAT6−/− skin (Figure 3K).

Because we measured gene expression in whole tissues, these changes reflect the combined contribution of epithelial, stromal, and immune cell populations and cannot be attributed exclusively to Tregs. Collectively, these findings show that STAT6 deficiency is associated with increased Treg accumulation in draining lymph nodes, a distinct distribution of Foxp3+ cells within papillomas, and elevated *Foxp3* expression in papilloma tissue. Together, these observations are consistent with an altered local immunoregulatory environment during DMBA/TPA-induced papilloma development.

### 3.4. STAT6 Deficiency Is Associated with Reduced CD8+ T-Cell Responses During Skin Papilloma Development

Given the increased Treg accumulation observed in STAT6−/− mice, we next investigated whether STAT6 deficiency was accompanied by changes in CD8+ T-cell responses. Cells from pooled draining axillary and inguinal lymph nodes from each mouse were analyzed by flow cytometry after 20 weeks of DMBA/TPA treatment. The gating strategy used to identify CD8+ T cells is shown in Figure 4A, and representative histograms of IFN-γ expression within this population are shown in Figure 4B.

The frequency of CD8+IFN-γ+ T cells was significantly increased in DMBA/TPA-treated WT mice compared with healthy controls (Figure 4C). In contrast, STAT6−/− mice did not exhibit a comparable increase following DMBA/TPA treatment. Analysis of absolute cell numbers similarly showed an increase in CD8+IFN-γ+ T cells in DMBA/TPA-treated WT mice, whereas this population was significantly reduced in DMBA/TPA-treated STAT6−/− mice compared with healthy control group and DMBA/TPA-treated WT mice (Figure 4D).

To assess the relative balance between CD8+ T cells and Tregs, we calculated the CD8/Foxp3 ratio using the flow-cytometry data. The ratio was significantly lower in DMBA/TPA-treated STAT6−/− mice than in treated WT mice (Figure 4E), indicating a relative shift toward Treg predominance. However, this ratio is influenced by both its CD8+ numerator and Foxp3+ denominator and therefore does not independently demonstrate reduced CD8+ T-cell abundance.

We next examined CD8+ cell infiltration within papillomas. Representative immunohistochemical images showed fewer CD8+ cells in STAT6−/− papillomas than in WT lesions (Figure 4F), and quantitative analysis confirmed a significant reduction in the number of infiltrating CD8+ cells per microscopic field in STAT6−/− papillomas (Figure 4G).

Finally, we evaluated *Ifng* mRNA expression in whole papilloma tissues and healthy skin. *Ifng* expression was significantly reduced in papillomas from both WT and STAT6−/− mice relative to their respective healthy controls (Figure 4H). No significant difference was detected between WT and STAT6−/− papillomas, although *Ifng* amplification was below the detection limit in most STAT6−/− samples. Because RNA was extracted from whole tissues, these measurements reflect the combined contribution of all cellular populations and cannot be attributed specifically to CD8+ T cells.

Collectively, these findings identify a consistent attenuation of CD8-associated immunity in STAT6−/− mice during DMBA/TPA-induced papilloma development. In contrast to WT mice, which mounted a clear increase in CD8+IFN-γ+ T cells following DMBA/TPA exposure, STAT6−/− mice exhibited reduced absolute numbers of this effector population, a markedly lower CD8/Foxp3 ratio, and significantly fewer infiltrating CD8+ cells within papillomas. The agreement between flow-cytometric and quantitative immunohistochemical analyses demonstrates that this phenotype extends from the draining lymph nodes to the local papilloma environment.

## 4. Discussion

Cutaneous squamous carcinogenesis is strongly influenced by chronic inflammation and alterations in local immune surveillance. Persistent epithelial damage promotes the release of inflammatory mediators, immune-cell recruitment, and remodeling of the tissue microenvironment [25]. In this context, the balance between antitumor effector responses and immunoregulatory mechanisms is critical in determining whether transformed keratinocytes are eliminated or allowed to progress. In the present study, STAT6 deficiency accelerated papilloma onset and markedly increased papilloma burden in a DMBA/TPA initiation–promotion model. This phenotype was associated with increased Foxp3+ Treg accumulation in skin-draining lymph nodes, a broader distribution of Foxp3+ cells within papillomas, reduced CD8+ cell infiltration, and attenuated CD8+IFN-γ+ responses in draining lymph nodes. Together, these findings associate STAT6 deficiency with increased susceptibility to papilloma development, and an altered balance between regulatory and CD8-associated immune responses.

The greater papilloma burden observed in STAT6-deficient mice at week 20 should be interpreted in the context of their earlier lesion onset. Because STAT6−/− mice developed papillomas earlier, lesions had more time to accumulate before the common experimental endpoint. Weekly mapping and tracking confirmed that the higher counts represented distinct, persistent papillomas rather than repeated counting of transient lesions. Nevertheless, the present analysis cannot distinguish whether the greater burden resulted from an increased rate of new papilloma formation, enhanced lesion persistence, the longer accumulation period associated with earlier onset, or a combination of these processes.

Previous studies using mice deficient in IL-4Rα, IL-13, or IL-13Rα1 have reported earlier onset or increased formation of DMBA/TPA-induced papillomas [17,18]. In particular, *Il13ra1*-deficient mice exhibited increased papilloma burden and Treg accumulation in skin-draining lymph nodes [18], showing phenotypic similarities to the changes observed in STAT6-deficient mice in the present study. However, this concordance does not establish that the phenotype caused by STAT6 deficiency is mediated through IL-13Rα1 or that the two knockout models share the same underlying mechanism. Our findings therefore establish an association between STAT6 deficiency and increased susceptibility to chemically induced papilloma development but do not identify the upstream signal responsible for this phenotype.

The protective role of STAT6 in this model contrasts with the protumoral functions reported for STAT6 in several other cancer types, highlighting the context-dependent nature of its activity [26]. STAT6 may influence both epithelial and immune compartments of the skin, including tissue homeostasis, barrier function, epithelial differentiation, chemokine expression, and immune-cell differentiation [27,28]. In keratinocytes, IL-4- and IL-13-mediated STAT6 activation can regulate epithelial differentiation programs and chemokine expression, including CCL26 [29]. Moreover, homeostatic IL-13 in healthy skin promotes the differentiation of dermal CD11b^lo^ migratory type 2 dendritic cells through a STAT6- and KLF4-dependent mechanism [30]. These observations suggest STAT6 may be involved in epithelial–immune communication but do not establish which upstream signal or cellular compartment drives the phenotype observed in our model. Because we used a global knockout model, this study cannot distinguish the relative contributions of STAT6 deficiency in keratinocytes, stromal cells, or individual immune-cell populations. Conditional knockout or bone-marrow chimera studies will be required to define the cellular compartments responsible for the observed phenotype.

Histopathological analysis further supported the association between STAT6 deficiency and features suggestive of squamous differentiation. Although DMBA/TPA treatment increased epidermal thickness in both genotypes, no significant difference was observed between WT and STAT6-/- mice. However, STAT6-deficient papillomas showed qualitative histopathological differences, including more pronounced acanthosis and hyperkeratosis, as well as keratin pearl-like structures. These observations may reflect differences in squamous differentiation but do not demonstrate malignant transformation. Interestingly, Ki-67 expression was higher in WT papillomas than in STAT6-deficient papillomas at week 20. This finding should not be interpreted as evidence of reduced papilloma development in STAT6-deficient mice, given their substantially earlier onset and greater papilloma burden. Because all tissues were collected at the same experimental endpoint, the analyzed lesions may have differed considerably in age and biological state. STAT6-deficient papillomas appeared earlier and may therefore have been older or more differentiated at tissue collection, whereas WT papillomas developed later and may have remained in a more actively proliferating phase. Nevertheless, this interpretation remains hypothetical because the endpoint analysis did not directly control for lesion age or differentiation status. Longitudinal histopathological and proliferative analyses at multiple time points, ideally comparing lesions of equivalent age and size, would be required to determine whether differences in Ki-67 expression reflect lesion age, differentiation state, or other genotype-dependent effects.

A central finding of this study is that STAT6 deficiency was associated with increased accumulation of Foxp3+ Tregs in skin-draining lymph nodes and papillomas. This observation is relevant because Tregs are key regulators of antitumor immunity and can suppress effector T-cell responses through multiple mechanisms, including inhibitory receptor pathways, cytokine-mediated suppression, metabolic competition, and modulation of antigen-presenting cell function [10]. In our model, STAT6-/- mice showed increased frequencies and absolute numbers of CD4+Foxp3+ cells in skin-draining lymph nodes, a broader distribution of Foxp3+ cells within papillomas, and increased PD-1 expression in Foxp3+ cells. Although PD-1 expression has been associated with activated tumor-associated Tregs [31,32], it does not by itself demonstrate enhanced suppressive capacity. Therefore, our findings identify increased representation of Foxp3+PD-1+ Tregs in STAT6-deficient mice, but functional suppression assays are needed to determine whether these cells exhibit greater suppressive activity or contribute directly to the increased papilloma burden.

The higher Foxp3 expression in STAT6−/− papillomas is consistent with the increased Treg frequency detected by flow cytometry and the broader distribution of Foxp3+ cells observed by immunohistochemistry. Nevertheless, because we measure gene expression in whole papilloma tissue, we cannot attribute this difference exclusively to Tregs. Tregs constitute a relatively small fraction of the total cellular population within these lesions; consequently, genotype-dependent transcriptional changes occurring specifically in Tregs may have been diluted by transcript expression from keratinocytes, stromal cells, and other infiltrating immune populations. Moreover, *Pdcd1* is not expressed exclusively by Tregs, further limiting the cell-specific interpretation of bulk-tissue measurements. Similarly, the reduced *Ctla4* expression observed in papillomas relative to healthy skin represents the net contribution of all cells within the tissue and cannot be interpreted as evidence of reduced CTLA-4 expression specifically in Tregs. Therefore, the cell-resolved flow-cytometric and immunohistochemical findings provide more direct evidence of genotype-associated differences in Treg accumulation, whereas the qPCR data describe the broader transcriptional environment of the papilloma. Analysis of sorted intralesional Tregs or single-cell approaches would be required to define their specific transcriptional profile.

The increased Treg accumulation observed in STAT6-deficient mice is consistent with previous evidence that STAT6 activation can antagonize Foxp3 induction. STAT6 has been shown to bind regulatory regions of the *Foxp3* locus and inhibit its transcription under Treg-polarizing conditions [12,15,33]. This evidence provides a biologically plausible context for the increased Treg frequency observed in our model. Nevertheless, the present experiments did not evaluate Treg differentiation, proliferation, survival, or recruitment and therefore do not establish the mechanism underlying their accumulation. Furthermore, because IL-4 and IL-13 concentrations, receptor activation, STAT6 phosphorylation, and STAT6 target-gene activation were not measured, the upstream signals involved cannot be determined from the present study.

Increased Treg accumulation in STAT6-deficient mice was accompanied by reduced CD8-associated IFN-γ responses, indicating an altered balance between regulatory and effector T-cell populations during papilloma development. The lower CD8/Treg ratio is consistent with a relative predominance of Tregs; however, interpret this ratio cautiously, as it may reflect increased Treg frequency rather than an absolute reduction in CD8+ T cells. In contrast, the reduced absolute number of CD8+IFN-γ+ cells in skin-draining lymph nodes and the lower infiltration of CD8+ cells within papillomas provide independent evidence of attenuated CD8-associated responses. Nevertheless, these measurements do not directly demonstrate impaired cytotoxic activity. Functional killing assays and measurements of additional cytotoxicity-associated molecules are needed to establish such an effect. Thus, our findings identify an immune profile characterized by increased Treg representation, reduced numbers of CD8+IFN-γ+ cells in skin-draining lymph nodes, and decreased CD8+ cell infiltration within papillomas in the absence of STAT6, without establishing a causal relationship between these immune alterations and the increased papilloma burden.

The role of IFN-γ in the DMBA/TPA model requires careful interpretation. Although IFN-γ is classically associated with antitumor immunity and cytotoxic T-cell activity, some studies have reported tumor-promoting effects of IFN-γ in chemically induced skin carcinogenesis, partly through the promotion of Th17-associated inflammation [34]. Conversely, other approaches using IFN-γ gene delivery have suggested inhibitory effects on DMBA/TPA-induced skin carcinogenesis [35]. These apparently divergent findings indicate that the biological effects of IFN-γ may depend on its timing, cellular source, inflammatory context, and downstream immune networks. In our study, we detected attenuated CD8+IFN-γ+ responses in draining lymph nodes of STAT6-deficient mice. In whole papilloma tissue, *Ifng* expression was reduced relative to healthy skin in both genotypes, but no significant difference was detected between WT and STAT6−/− papillomas. Thus, the flow-cytometric findings demonstrate a genotype-associated difference within the CD8+ T-cell compartment of skin-draining lymph nodes, whereas the bulk-tissue qPCR results do not support a genotype-dependent difference in overall *Ifng* expression within papillomas.

This study has several limitations. First, using global STAT6-deficient mice does not allow us to distinguish whether the phenotype is mediated by epithelial cells, immune cells, stromal cells, or a combination of these compartments. Second, although our data show increased Treg accumulation and PD-1 expression, we did not permorm functional suppression assays to determine the suppressive activity of Tregs from STAT6-deficient mice. Third, the present study cannot establish whether Treg accumulation results from altered differentiation, proliferation, survival, recruitment, or a combination of these processes. Fourth, we did not comprehensively characterized additional immune populations, including dendritic cells, macrophages, eosinophils, neutrophils, and Th17 cells which may contribute to the observed phenotype. The 20-week experimental protocol was designed to evaluate papilloma development and did not assess malignant conversion to invasive squamous cell carcinoma. Longer follow-up and dedicated histopathological analyses will therefore be required to determine whether STAT6 deficiency also influences malignant progression. Finally, because WT and STAT6−/− mice were maintained as separate homozygous colonies rather than as littermate controls, potential breeding-history-associated differences cannot be completely excluded, despite matching genetic background, age, sex, and husbandry conditions. Figure 5 summarizes the potential mechanisms by which STAT6 deficiency may be associated with papilloma development.

## 5. Conclusions

This study demonstrates that STAT6 deficiency is associated with earlier onset and markedly increased burden of DMBA/TPA-induced skin papillomas. This phenotype was accompanied by keratinization-related histopathological alterations and a distinct immune profile characterized by increased accumulation of Foxp3+PD-1+ Tregs, reduced numbers of CD8+IFN-γ+ cells in skin-draining lymph nodes, and decreased CD8+ cell infiltration within papillomas.

Together, these findings establish a previously unrecognized association between STAT6 deficiency, susceptibility to chemically induced skin papilloma development, and an altered balance between regulatory and CD8-associated immune responses. They also highlight the context-dependent role of STAT6 in cutaneous tumor immunity and underscore the importance of considering tissue-specific immune effects when evaluating therapeutic strategies aimed at modulating STAT6 signaling.

## Figures and Tables

**Figure 1:**
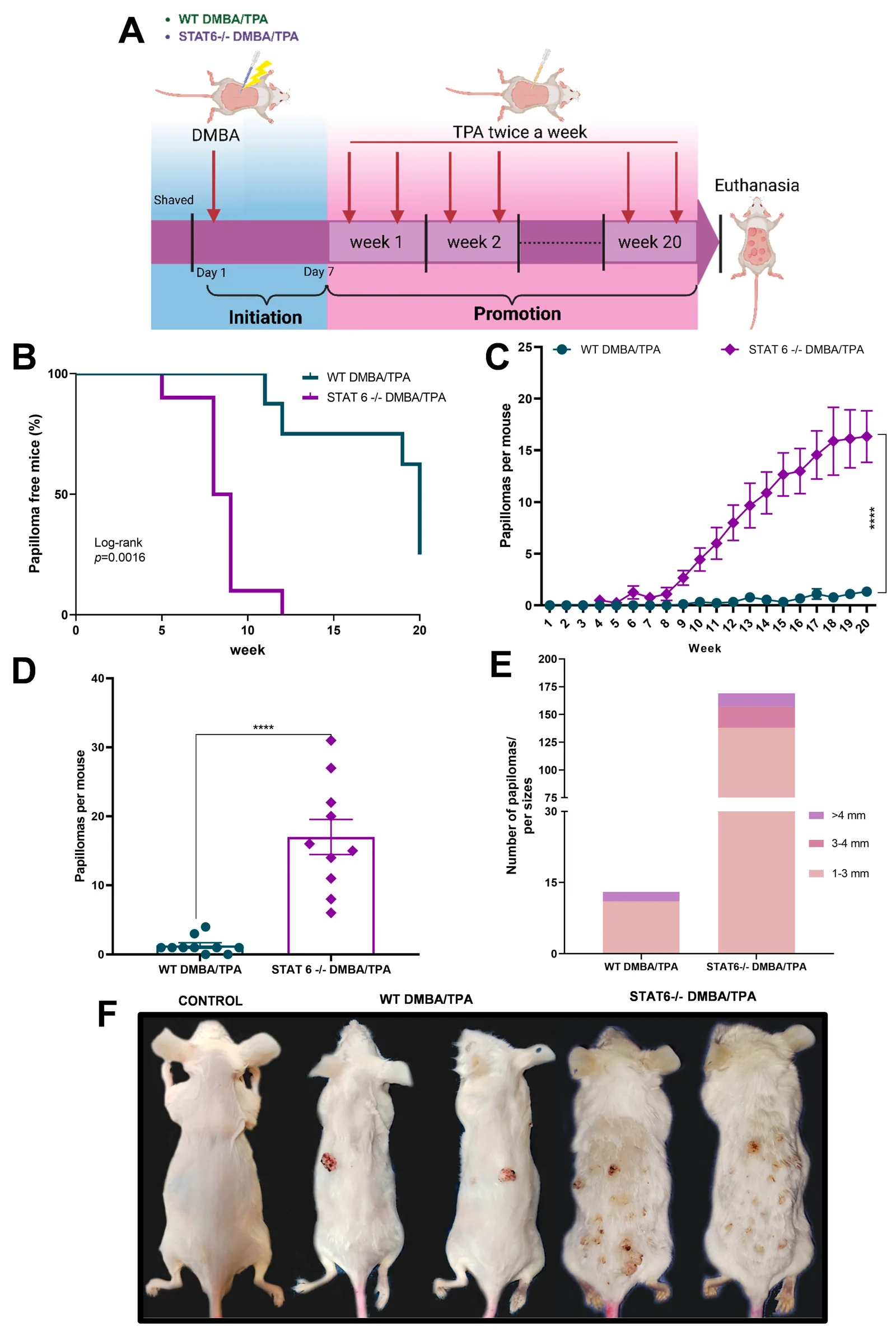
STAT6 deficiency accelerates DMBA/TPA-induced skin papilloma development. (**A**) Timeline of the two-stage DMBA/TPA initiation–promotion protocol. Red arrows indicate the time points of DMBA or TPA application. (**B**) Kaplan–Meier curves showing papilloma-free probability, defined as the time to the first detection of a lesion larger than 1 mm at two consecutive weekly examinations. Papilloma onset differed significantly between WT and STAT6−/− mice (log-rank [Mantel–Cox] test, *p* = 0.0016). (**C**) Longitudinal papilloma counts per mouse. Differences between genotypes were evaluated using generalized linear mixed-effects model with a Poisson distribution, with genotype, week, and their interaction as fixed effects and mouse identity as a random intercept. Model-derived pairwise comparisons were performed at individual weeks using estimated marginal means. STAT6−/− mice showed higher expected papilloma counts than WT mice (**** *p* < 0.0001). (**D**) Papilloma counts per mouse at week 20. The model-derived contrast indicated a higher expected papilloma count in STAT6−/− mice than in WT mice (KO/WT ratio = 19.8; z = 6.931; *p* < 0.0001). (**E**) Descriptive distribution of papillomas according to diameter category (1–3 mm, 3–4 mm, and >4 mm) at week 20. Bars represent the total number of papillomas recorded across all 10 mice within each genotype; no inferential statistical analysis was performed. (**F**) Representative photographs of the dorsal skin at week 20. Data in panels (**C**,**D**) are presented as mean ± SEM; each point in panel (**D**) represents one mouse. Panel (**E**) shows aggregate papilloma counts without error bars or statistical comparisons. All animals were included in the analyses, including those that remained papilloma-free. *n* = 10 mice per genotype from two independent experiments.

**Figure 2:**
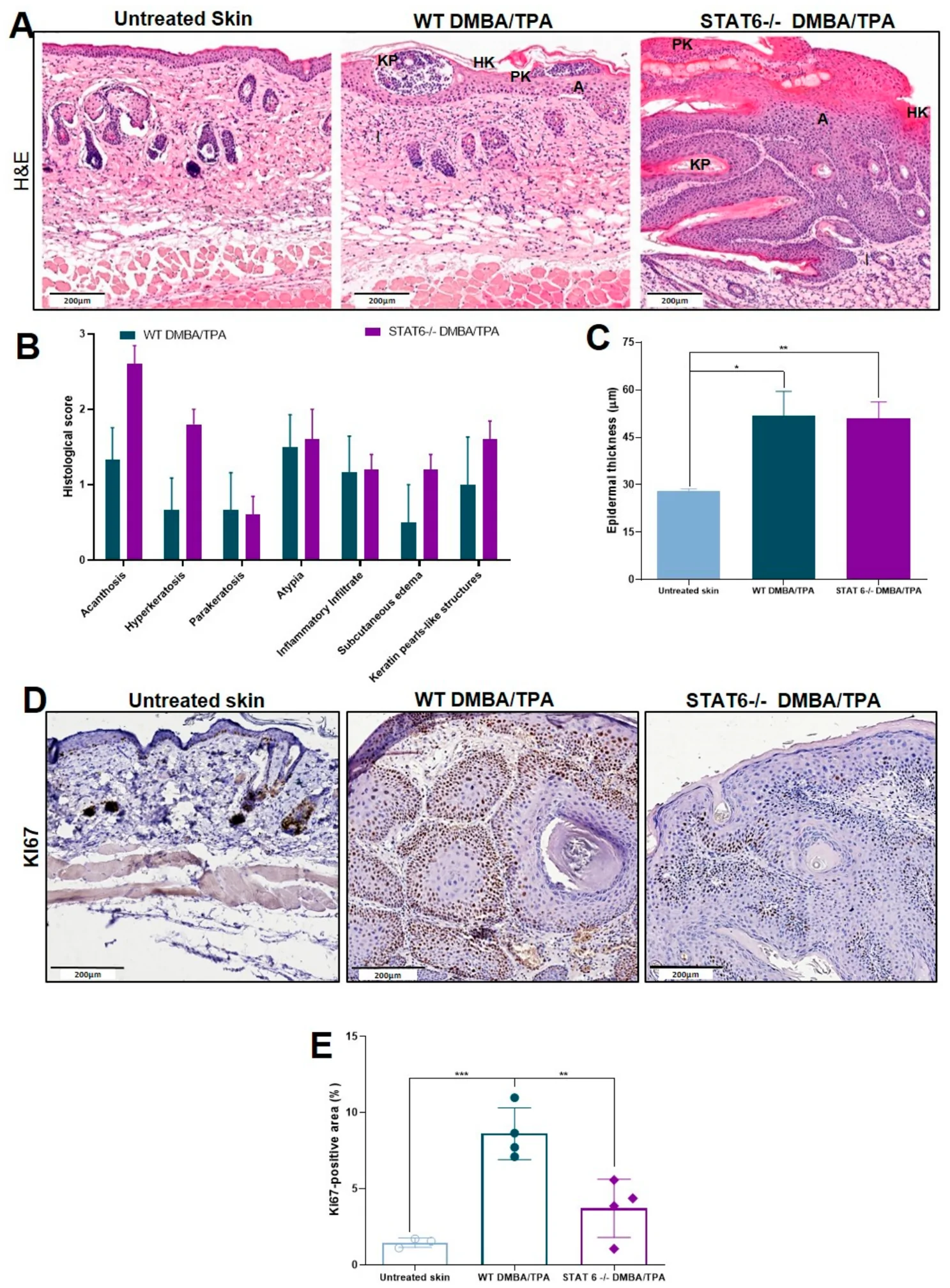
STAT6 deficiency is associated with distinct histopathological alterations during DMBA/TPA-induced skin papilloma development. (**A**) Representative hematoxylin and eosin (H&E)-stained sections of healthy skin and DMBA/TPA-treated skin from WT and STAT6−/− mice. A, acanthosis; HK, hyperkeratosis; PK, parakeratosis; KP, keratin pearl; I, inflammatory infiltrate. (**B**) Descriptive semiquantitative scores for the indicated histopathological features. Field- and lesion-level scores were averaged to obtain one value per mouse. Bars show the mean mouse-level score for each genotype; no inferential statistical comparisons were performed. (**C**) Epidermal thickness in healthy and DMBA/TPA-treated skin. (**D**) Representative Ki-67 immunostaining in healthy and DMBA/TPA-treated skin. (**E**) Percentage of Ki-67-positive area determined by color segmentation in ImageJ. Three microscopic fields per mouse were averaged to obtain one value per animal. Data are presented as mean ± SEM. Panels (**B**,**C**) include 10 mice per genotype from two independent experiments. Panel (**E**) shows four mice per group from one representative experiment; comparable staining patterns were observed in two independent experiments. Each data point represents one mouse. Scale bars = 200 µm. Data in panels (**C**,**E**) were analyzed using one-way ANOVA followed by Tukey’s multiple-comparisons test. * *p* < 0.05, ** *p* < 0.01, and *** *p* < 0.001.

**Figure 3:**
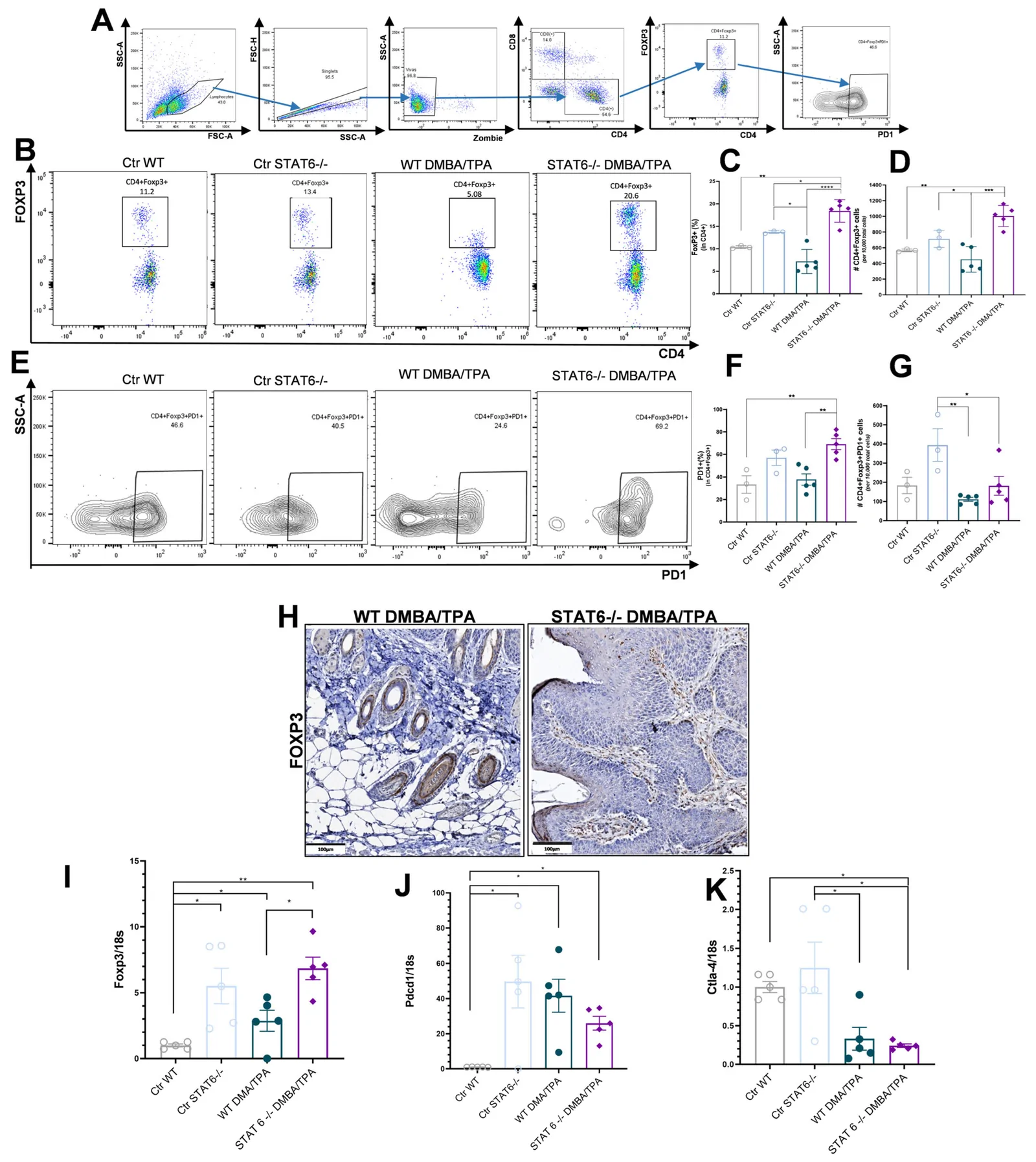
STAT6 deficiency is associated with increased Treg accumulation in draining lymph nodes and a distinct distribution of Foxp3+ cells within papillomas. (**A**) Flow-cytometry gating strategy used to identify CD4+Foxp3+ Tregs and CD4+Foxp3+PD−1+ Tregs in pooled draining axillary and inguinal lymph nodes from each mouse. (**B**) Representative dot plots showing CD4+Foxp3+ Tregs in healthy WT and STAT6−/− mice and in WT and STAT6−/− mice after 20 weeks of DMBA/TPA treatment. (**C**,**D**) Frequency and absolute number, respectively, of CD4+Foxp3+ cells. (**E**) Representative dot plots showing PD−1 expression within the CD4+Foxp3+ population. (**F**,**G**) Frequency and absolute number, respectively, of CD4+Foxp3+PD−1+ Tregs. (**H**) Representative Foxp3 immunohistochemistry in papilloma tissues from WT and STAT6−/− mice after 20 weeks of DMBA/TPA treatment. (**I**–**K**) Relative mRNA expression of *Foxp3* (**I)**, *Pdcd1* (**J**), and *Ctla4* (**K**) in healthy skin and papilloma tissues collected after 20 weeks of DMBA/TPA treatment. Data are presented as mean ± SEM, and each data point represents one mouse. Data in panels (**C**,**D**,**F**,**G**,**I**–**K**) were analyzed using one-way ANOVA followed by Tukey’s multiple-comparisons test. For flow cytometry, *n* = 3 mice per healthy control group and *n* = 5 mice per DMBA/TPA-treated group. For qPCR, *n* = 5 mice per group. Results are shown from one representative experiment of two independent experiments. * *p* < 0.05, ** *p* < 0.01, *** *p* < 0.001 and **** *p*< 0.0001.

**Figure 4:**
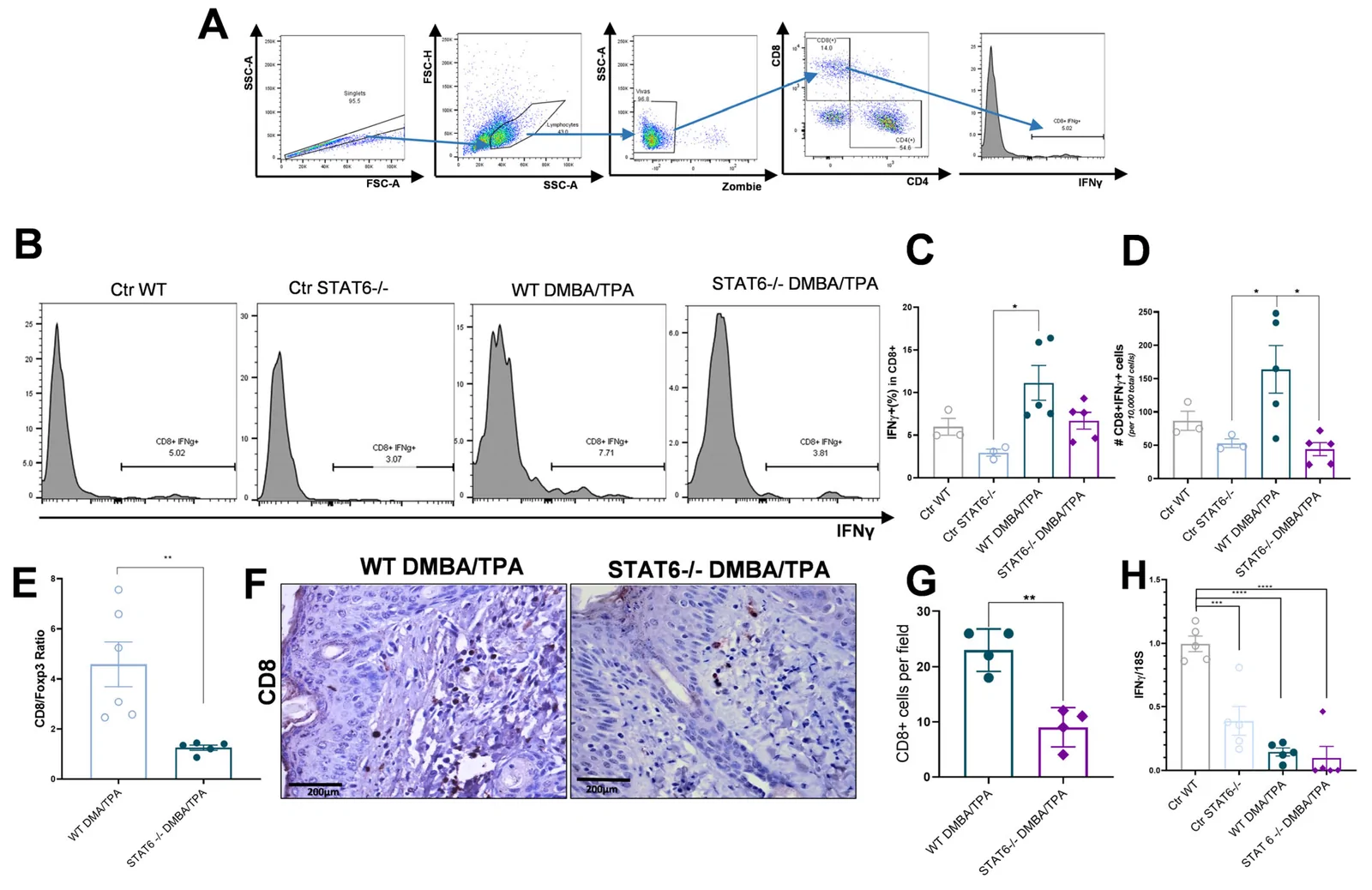
STAT6 deficiency is associated with attenuated CD8+ T-cell responses in draining lymph nodes and reduced CD8+ cell infiltration within papillomas. (**A**) Representative flow-cytometry gating strategy used to identify CD8+ T cells in pooled draining axillary and inguinal lymph nodes from each mouse. (**B**) Representative histograms showing IFN-γ expression within the CD8+ T-cell population from healthy WT and STAT6−/− mice and from WT and STAT6−/− mice after 20 weeks of DMBA/TPA treatment. (**C**,**D**) Frequency and absolute number, respectively, of CD8+IFN-γ+ T cells. (**E**) CD8/Foxp3 ratio calculated from the flow-cytometry data. Axillary and inguinal lymph nodes were pooled within each mouse but not across animals; therefore, each data point represents one mouse. (**F**) Representative CD8 immunohistochemistry in papilloma tissues from WT and STAT6−/− mice. (**G**) Quantification of CD8+ cells within papillomas. Three microscopic fields were evaluated per mouse and averaged to obtain one biological value per animal. (**H**) Relative *Ifng* mRNA expression in healthy skin and papilloma tissues collected after 20 weeks of DMBA/TPA treatment. Data are presented as mean ± SEM, and each data point represents one mouse. Panels (**C**–**E**,**H**)were analyzed using one-way ANOVA followed by Tukey’s multiple-comparisons test. Panel G was analyzed using an unpaired two-tailed Student’s t-test. For flow cytometry, *n* = 3 mice per healthy control group and *n* = 5 mice per DMBA/TPA-treated group. For immunohistochemistry, *n* = 4 mice per group. For qPCR, *n* = 5 mice per group. Scale bars = 200 µm. Results shown are from one representative experiment. * *p* < 0.05, ** *p* < 0.01, *** *p*< 0.001, **** *p* < 0.0001.

**Figure 5:**
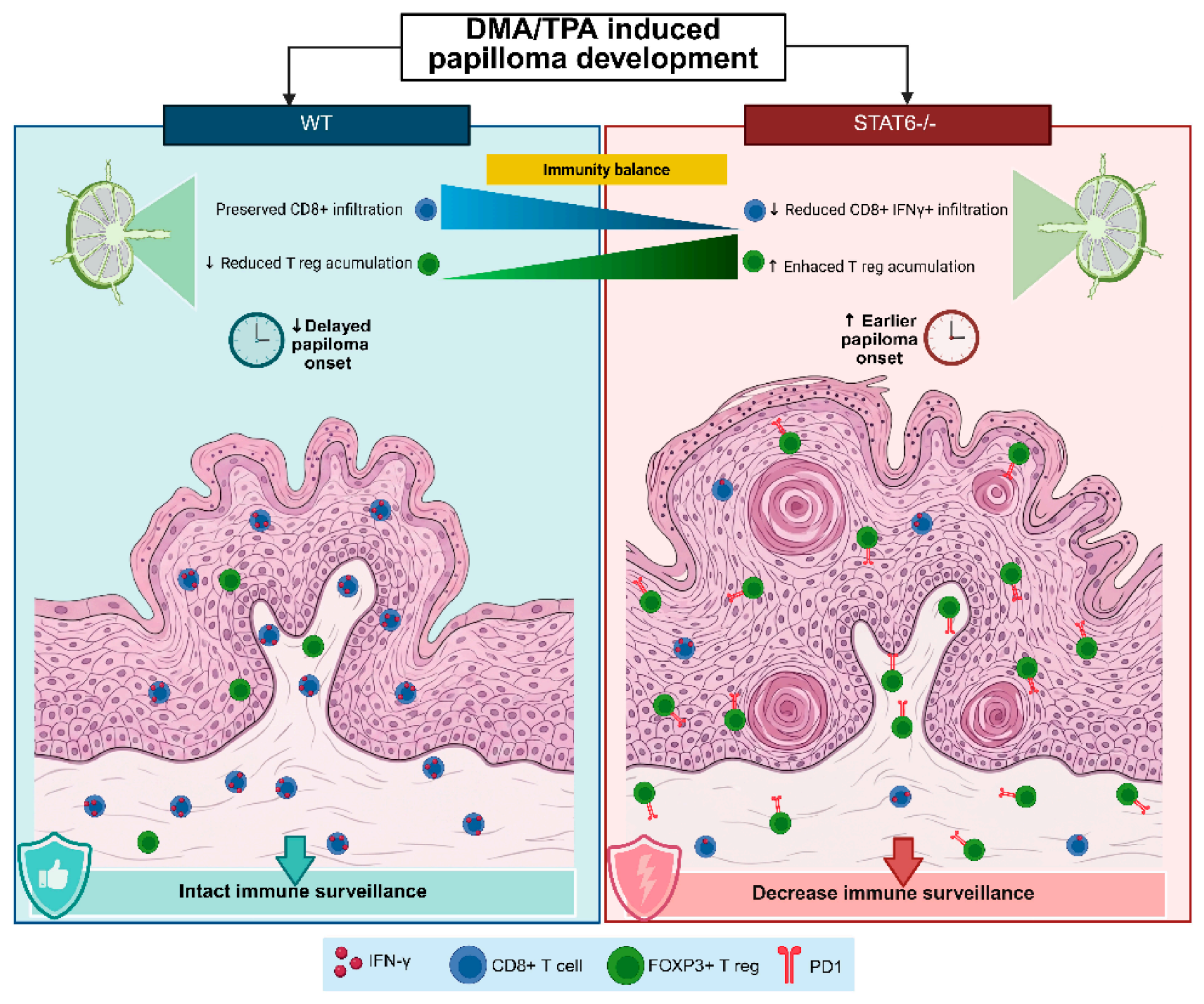
Proposed model of the immune alterations associated with STAT6 deficiency during DMBA/TPA-induced skin papilloma development. Compared with WT mice, STAT6−/− mice developed papillomas earlier and exhibited a greater papilloma burden. This phenotype was associated with increased accumulation of Foxp3+PD-1+ Tregs and attenuated CD8+IFN-γ+ responses in skin-draining lymph nodes, together with reduced CD8+ cell infiltration and a broader distribution of Foxp3+ cells within papillomas. These findings define an altered immune profile characterized by greater Treg representation and reduced CD8-associated responses in STAT6−/− mice.

## Data Availability

The original contributions presented in this study are included in the article. Further inquiries can be directed to the corresponding author.

## Appendix A

**Table A1:** Primer sequences.

Gene	Primer Forward	Primer Reverse
*ifng*	AGGAACTGGCAAAAGGATGGTG	GCTGGACCTGTGGGTTGTT
*foxp3*	GCGTAAGCAGGGCAATAGAGG	GCATGAGGTCAAGGGTGA
*pdcd1*	GCAGTTGAGCTGGCAATCAG	GTGAAGGTGGCATTTGCTCC
*ctla4*	TACTCTGCTCCCTGAGGACC	CTGCTAGCCAACACCACTGA

**Table A2:** Semiquantitative scale used for histopathological assessment.

Score	Definition *
0	Feature absent
1	Mild or focal presence
2	Moderate or multifocal presence
3	Severe or diffuse presence

* The scale was independently applied to acanthosis, hyperkeratosis, parakeratosis, atypia, inflammatory infiltrate, subcutaneous edema, keratin pearl-like structures, vasculitis, and adnexal damage.
